# Anti-RAINBOW dye-specific antibodies as universal tools for the visualization of prestained protein molecular weight markers in Western blot analysis

**DOI:** 10.1038/srep31363

**Published:** 2016-08-17

**Authors:** Stefan Schüchner, Peter Andorfer, Ingrid Mudrak, Egon Ogris

**Affiliations:** 1 Department of Medical Biochemistry, Max F. Perutz Laboratories, Medical University of Vienna, Dr. Bohr-Gasse 9, A-1030 Vienna, Austria

## Abstract

Western blotting is one of the most widely used techniques in molecular biology and biochemistry. Prestained proteins are used as molecular weight standards in protein electrophoresis. In the chemiluminescent Western blot analysis, however, these colored protein markers are invisible leaving researchers with the unsatisfying situation that the signal for the protein of interest and the signal for the markers are not captured simultaneously and have to be merged in an error-prone step. To allow the simultaneous detection of marker proteins we generated monoclonal antibodies specific for the protein dyes. To elicit a dye rather than protein specific immune response we immunized mice sequentially with dye-carrier protein complexes, in which a new carrier protein was used for each subsequent immunization. Moreover, by sequentially immunizing with dye-carrier protein complexes, in which different but structurally related dyes were used, we could also generate an antibody, termed anti-RAINBOW, that cross-reacted even with structurally related dyes not used in the immunizations. Our novel antibodies represent convenient tools for the simultaneous Western blot detection of commercially available prestained marker proteins in combination with the detection of any specific protein of interest. These antibodies will render obsolete the anachronistic tradition of manually charting marker bands on film.

The most widely used method for the analysis of proteins is sodium dodecyl sulfate (SDS)-polyacrylamide gel electrophoresis (PAGE)^1^, which is often followed by transferring the proteins to a membrane, where the proteins get immobilized and detected with antibodies, generally referred to as Western blot analysis^2^. To estimate the relative molecular weight of a specific protein, protein molecular weight markers are separated side-by-side with the protein sample. Almost all of the commercially available molecular weight markers consist of proteins prestained with vinyl sulfone dyes, also known under their trademark name as Remazol^®^ dyes, which provide visible reference points for the proteins of interest^3^,^4^,^5^. These proteins of interest, however, have to be visualized by specific antibodies that are coupled to fluorophores or enzymes catalyzing a chemiluminescent reaction. The most widely used enzyme for Western blot detection is horseradish peroxidase (HRP), which catalyzes the chemiluminescent oxidation of luminol. The emitted light is detected either on X-ray films or with the help of CCD-based camera systems. The major advantage of chemiluminescence over fluorescence detection is the signal amplification due to the enzyme catalyzed reaction, allowing the detection of minute amounts of the target protein. The prestained molecular weight marker proteins, however, are not detected by the chemiluminescent reaction and are therefore not displayed on the X-ray film, making it necessary to manually chart the protein marker bands on the film (or to overlay the CCD camera captured picture of the emitted light with the one of the stained marker captured under daylight) in order to estimate the molecular weight of the detected protein bands. This process not only seems anachronistic in an otherwise high-tech research field but is intrinsically prone to human error, as the film is fitted to the membrane and has to be perfectly positioned to accurately copy the marker bands: first, reference points are often lacking since the contours of the membrane are not visible on the film, and second, any inaccuracy of the experimenter in mapping the shapes of the marker bands may directly affect data interpretation. This problem has been addressed several times, but all of the currently available systems have major disadvantages and restrictions that limit their usage. The so-called Optiblot Luminol Pen (Abcam) is easy to use, but still requires the manual labeling of protein marker bands. Protein molecular weight markers coupled to fluorescent dyes can be directly detected by Western blot analysis, but require expensive scanner equipment (e.g. LI-COR Odyssey, or GE Healthcare Typhoon). Other marker proteins were engineered to contain immunoglobulin G (IgG) binding sites (e.g. MagicMark^TM^ XP Western Protein Standard, SuperSignal Molecular Weight Protein Ladder, both Life Technologies), which allow their detection with standard secondary antibodies; however, these are species-specific IgG binding sites and therefore different protein marker ladders have to be matched to the appropriate secondary antibody used. Moreover due to their intrinsic binding affinities for the Ig Fc domain also primary antibodies directed against the desired target are bound by these markers reducing their availability for antigen detection. Cell Signaling Technology offers biotinylated marker proteins that are detected with an anti-biotin-HRP coupled antibody, but this antibody cross-reacts with any biotinylated proteins in the cell lysate, which limits its usage to those cell types that do not contain biotinylated proteins. Similar systems based on HRP-coupled StrepTactin/streptavidin are also available (WesternC^TM^, Bio-Rad; or Chemi-Lumi One Marker, Nacalai Tesque). All these approaches use marker proteins that were modified for their detection in Western blot analysis, a strategy, which restricts the researcher to a particular marker product from a specific manufacturer. Up to now, however, there is no general detection tool for prestained markers. To circumvent these limitations, we have developed a series of mouse monoclonal antibodies for the Western blot detection of Remazol dye-stained marker proteins. Our antibodies are highly specific for Remazol dye stained proteins, recognize all Remazol dye prestained protein markers tested and do not cross-react with unstained cellular proteins, making them ideal and versatile tools for the detection of protein molecular weight marker bands by Western blot analysis.

## Results

**Figure 1:**
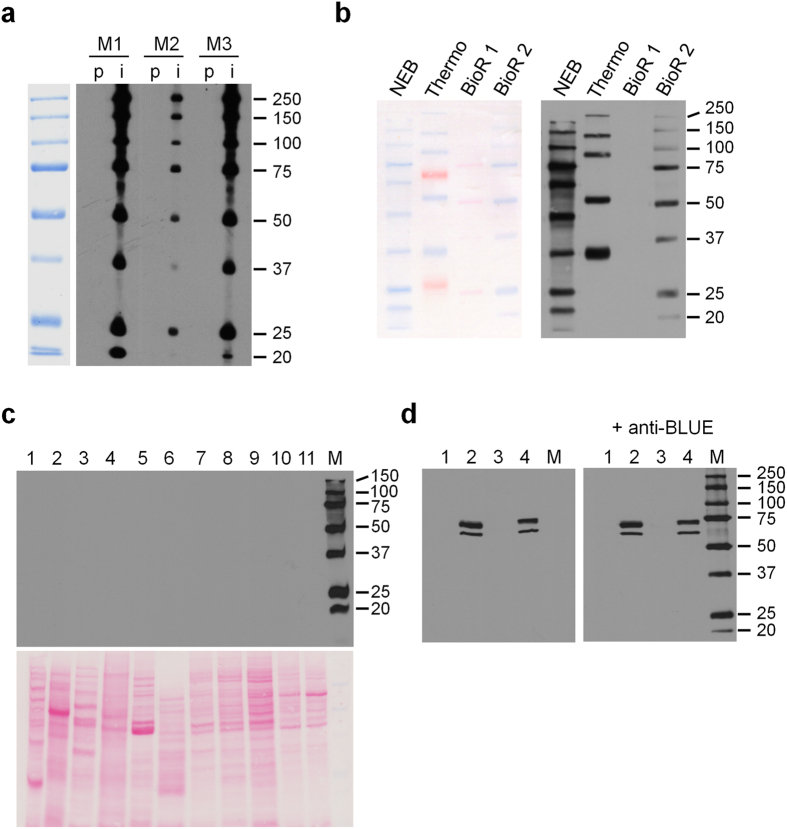
A Remazol Brilliant Blue R specific monoclonal antibody detects Blue prestained molecular weight markers. (**a)** Presence of reactive antibodies against Bio-Rad Precision Plus All Blue protein marker in mouse sera. Marker proteins were separated by preparative 10% SDS-PAGE (left lane) and sera were tested with a Miniblotter 28 channel unit. M1, M2, M3: mouse 1, 2, 3; p: preimmune serum; i: immune serum after 3^rd^ immunization. For better visualization, 5× more protein marker was loaded on the colorimetric lane than on the lanes used for Western blot analysis with the mouse sera. (**b)** Detection of commercial prestained protein molecular weight markers by the anti-BLUE monoclonal antibody. NEB: New England Biolabs Blue Prestained Protein Standard, broad range (P7706; 1 μl); Thermo: ThermoScientific Page Ruler Plus (26619; 1 μl); BioR1: Precision Plus Protein Unstained Protein Standard (161-0363; 2 μl); BioR2: Bio-Rad Precision Plus Protein All Blue Prestained Protein Standard (161-0373; 1 μl) separated by 10% SDS-PAGE and transferred to nitrocellulose membrane (left panel). The membrane was then incubated as described in Materials & Methods with anti-BLUE (right panel). (**c)** anti-BLUE does not cross-react with unstained proteins from whole cell lysates. 1: *E*. *coli*; 2: *A*. *thaliana*; 3: *S*. *cerevisiae*; 4: *C*. *elegans*; 5: *D*. *melanogaster*; 6: Chicken follicle; 7: CHO, Chinese hamster ovary; 8: Rat1, rat fibroblasts; 9: N2a, mouse neuroblastoma; 10: CV1, Green monkey epithelial; 11: Hela, human epithelial; M: Bio-Rad Precision Plus Protein All Blue (1 μl). Nitrocellulose membrane stained with Ponceau S (lower panel). The membrane was then incubated as described in Materials & Methods with anti-BLUE (upper panel). (**d)** Simultaneous detection of protein marker with detection of Lamin A/C. One of two identical blots containing the indicated lysates was incubated with anti-Lamin A/C antibody, clone 4C11, alone (left panel) or in combination with anti-BLUE (right panel). 1: HAP1 LMNA^−^, near-haploid human adherent cell line with a fibroblast-like morphology, lacking the LMNA gene; 2: HAP1 wildtype/control cells; 3: MEF LMNA^−/−^, mouse embryo fibroblasts lacking the LMNA gene; 4: MEF wildtype/control cells; M: Bio-Rad Precision Plus Protein All Blue (1 μl).

Most prestained protein molecular weight markers consist of blue stained proteins with defined molecular weights. To selectively elicit an immune response rather against the blue dye than the unstained molecular weight marker proteins, we developed an immunization strategy, in which mice were immunized consecutively with three different blue-stained proteins, namely the 25 kDa, the 50 kDa and the 75 kDa marker proteins, from the Precision Plus Protein^TM^ All Blue Prestained Protein Standard (Bio-Rad). Screening of mouse sera by Western blot against the Precision Plus Protein marker revealed, that all antisera not only detected the 25 kDa, the 50 kDa and the 75 kDa marker proteins but also other Precision Plus Protein^TM^ marker proteins that had not been used for immunization (Fig. 1a) indicating the presence of dye-specific antibodies. Although not disclosed by Bio-Rad, the dye used to stain the proteins of the Precision Plus Protein marker is most likely Remazol Brilliant Blue R (RBBR), an anthraquinone dye used in textile industry that under basic conditions covalently binds to reactive amino-, hydroxy- and sulfhydryl-groups. Besides their use to color textiles, Remazol dyes have also been applied in a variety of molecular biological techniques for the staining of proteins as well as non-proteinogenic molecules^4^,^6^,^7^,^8^,^9^,^10^. To show that our immunization strategy indeed can generate RBBR-specific antibodies we coupled RBBR to BSA, ADH and lysozyme, as described by Compton *et al*.^5^, and immunized mice according to the above described strategy; first with RBBR-BSA, then with RBBR-ADH and finally with RBBR-lysozyme. This approach again evoked a strong immune response against RBBR-stained proteins and only a weak response against the unstained carrier proteins (Supplementary Figure 1). Importantly, all Precision Plus prestained marker proteins were recognized very well by the antisera (Supplementary Figure 2). Following a final boost with a mixture of the three immunization proteins, we generated RBBR-specific monoclonal antibodies from these mice, generally termed anti-BLUE, and characterized their immunoblotting properties. Anti-BLUE clone 2D2-F11, which was isolated from a mouse immunized with prestained Precision Plus Protein marker proteins, detected not only the Bio-Rad protein marker bands but also blue prestained marker proteins from different companies (Fig. 1b). Unstained marker proteins (Fig. 1b, BioR1 lane, non-covalently stained with Ponceau S) or marker proteins prestained with different dyes (Fig. 1b, red stained marker bands in Thermo lane) were not detected indicating its specificity for Remazol Brilliant Blue R. Similar results were achieved with other clones including those isolated from mice immunized with RBBR-proteins (e.g. clone 6F4-F6, data not shown). Interestingly, the antibody did not bind to the free Remazol dye as tested by dot blot analysis (Supplementary Figure 3). A key requirement for the detection of prestained marker protein bands in a standard Western blot analysis is the lack of cross-reactivity of the anti-BLUE antibody with any unstained proteome. Therefore, we tested the antibody against whole cell lysates from a wide variety of species, including mouse, human, yeast and *E*. *coli*, and did not detect any cross-reactivity (Fig. 1c). This exquisite specificity for RBBR stained proteins was further confirmed in a series of Western blot experiments, in which the anti-BLUE antibody was applied in combination with a variety of primary antibodies, including a mouse monoclonal antibody against lamin A/C as well as mono- and polyclonal antibodies against different mammalian or yeast targets (Fig. 1d and Supplementary Figures 4 and 11). In none of these experiments we observed differences in the banding pattern between incubation with primary antibody alone or in combination with the anti-BLUE antibody.

**Figure 2:**
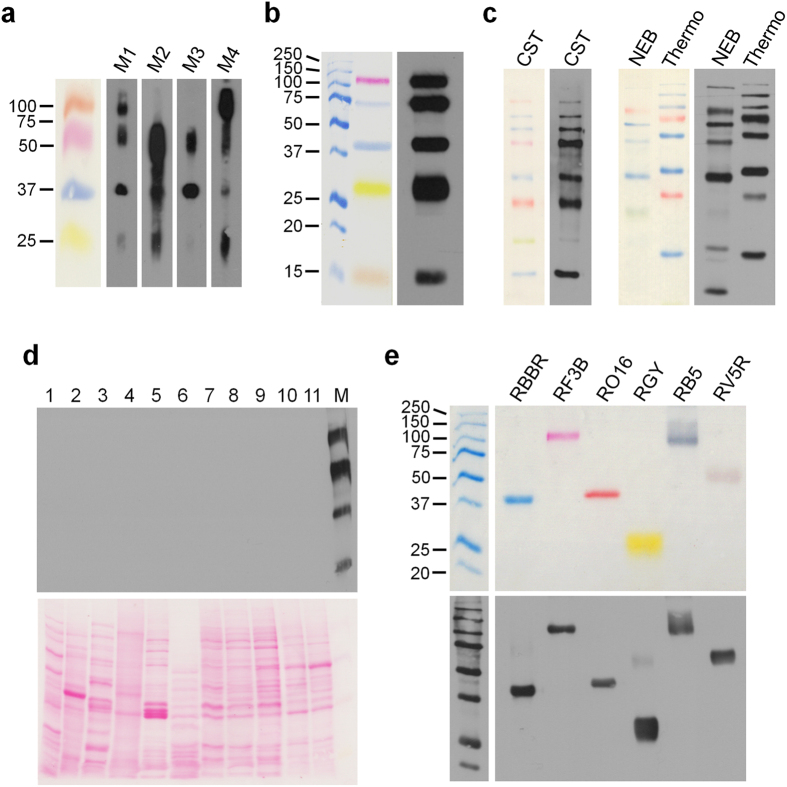
Anti-RAINBOW antibody detects multi-colour Remazol prestained molecular weight markers. (**a**) Presence of antibodies against four Remazol dyes in mouse sera. Immune sera of mice (3^rd^ bleed) immunized with different prestained proteins were tested with a Miniblotter 28 channel unit against Remazol dye stained phosphorylase b (orange), γ-globulin heavy chain (red), ADH (blue), and γ-globulin light chain (yellow). M1, M2, M3, M4: mouse 1, 2, 3, 4. 20× more dye stained proteins were loaded on the colorimetric marker lane than on the lanes used for Western blot analysis with the mouse sera. (**b**) The anti-RAINBOW monoclonal antibody shows equal detection of Remazol-dye stained proteins: phosphorylase b (red), BSA and ADH (blue), chymotrypsin (yellow) and lysozyme (orange). 10× more dye stained proteins were loaded on the colorimetric lane shown in the left panel than on the lane incubated with anti-RAINBOW antibody. (**c)** anti-RAINBOW monoclonal antibody detects all marker bands in a variety of commercial multicolor protein marker mixtures. CST: Cell Signaling Technology, Color-coded Prestained Protein Marker, High Range (12949P); NEB: New England Biolabs, ColorPlus Prestained Protein Marker (P7709); Thermo: Thermo Scientific: PageRuler Plus Prestained Protein Ladder (26619). 5× more of each protein marker mixture was loaded on the colorimetric lanes with the visible marker bands than on the lanes incubated with anti-RAINBOW antibody. (**d**) anti-RAINBOW monoclonal antibody does not cross-react with unstained cellular proteins. 1: *E*. *coli*; 2: *A*. *thaliana*; 3: *S*. *cerevisiae*; 4: *C*. *elegans*; 5: *D*. *melanogaster*; 6: Chicken follicle; 7: CHO, Chinese hamster ovary; 8: Rat1, rat fibroblasts; 9: N2a, mouse neuroblastoma; 10: CV1, Green monkey epithelial; 11: Hela, human epithelial; M: homemade prestained rainbow marker (RF3B-phosphorylase b, RBBR-BSA, RO16-ADH, RGY-γ-globulin light chain). (**e**) anti-RAINBOW monoclonal antibody cross-reacts with Remazol dyes not used for immunization. Lane 1: RBBR-ADH (blue), lane 2: RF3B-phosphorylase b (red), lane 3: RO16-ADH (orange), lane 4: RGY-γ-globulin light chain (yellow), lane 5: RB5-phosphorylase b (black), lane 6: RV5R-γ-globulin heavy chain (violet). 5× more Bio-Rad marker proteins and 10× more of the Remazol stained proteins were loaded on the upper blot (colorimetric image) than on the lower blot incubated with anti-RAINBOW antibody.

With the exception of the green stained marker protein, the anti-BLUE did not detect marker proteins stained with other Remazol dyes that are constituents of commercially available “rainbow marker” ladders. These reactive textile dyes, like Remazol Red F3B (RRF3B), Remazol Orange 16 (RO16), and Remazol Golden Yellow RNL (RGY), share an aromatic ring system and the active vinyl sulfone group^5^ (Supplementary Figure 5). Building on our immunization strategy used to generate the anti-BLUE we reasoned that it should be possible to generate antibodies that recognize an epitope common to the different Remazol dyes and therefore cross-detect all rainbow colors of marker proteins. Thus, we immunized mice sequentially with different combinations of four different carrier proteins stained with the four different Remazol dyes RBBR (blue), RRF3B (red), RO16 (orange) and RGY (yellow) with the hypothesis that this approach will lead to the maturation of antibodies specific for an epitope shared between these dyes (Fig. 2a). While all mice developed an immune response towards several of the colored proteins, the ability to recognize the different colors varied very much between antisera, with only one antiserum recognizing the differently colored marker proteins in a more balanced manner. This ability, however, could be caused by the presence of several dye-specific antibodies in the antiserum rather than by antibodies cross-reacting with different Remazol dyes. To distinguish between these possibilities we generated monoclonal antibodies and screened for dye cross-reactive antibodies. After a final boost containing a mixture of all four protein/dye conjugates, we isolated a monoclonal antibody, termed anti-RAINBOW, that indeed cross-reacted with comparable affinity with the four different Remazol dye immunogens coupled to proteins (Fig. 2b) but in addition also cross-reacted with two Remazol dyes, Remazol Brilliant Violet and Remazol Reactive Black 5 that had not been used (Fig. 2e and Supplementary Figure 6) for immunizations pointing to a common epitope in these Remazol dyes. Indeed, the pink marker bands of the Bio-Rad Precision Plus Protein^TM^ WesternC^TM^ Standard, which according to the manufacturer are not Remazol dye stained proteins (the exact nature of the dye was not disclosed), were not detected by the anti-RAINBOW antibody. Nevertheless these pink bands showed up as “negative” stain in the chemiluminescent detection, thereby again making possible the identification of the respective protein marker bands (Supplementary Figure 7). Moreover, the anti-RAINBOW antibody detected the blue stained bands of this marker with superior sensitivity and much lesser background than Bio-Rad’s own StrepTactin-HRP detection system (Supplementary Figure 7). With this single exception, the anti-RAINBOW antibody detected all marker proteins in commercially available “RAINBOW markers” tested including green marker bands while at the same time lacking cross-reactivity with unstained proteins in whole cell lysates of a variety of species (Fig. 2c,d). While our data did not indicate any cross-reactivity of the anti-BLUE/anti-RAINBOW antibodies with unstained proteins, we cannot rule out the possibility of cross-reactivity in other materials, e.g. in different tissue types than the tested ones. Thus, proper controls should always be included when using these new tools for the first time.

**Figure 3:**
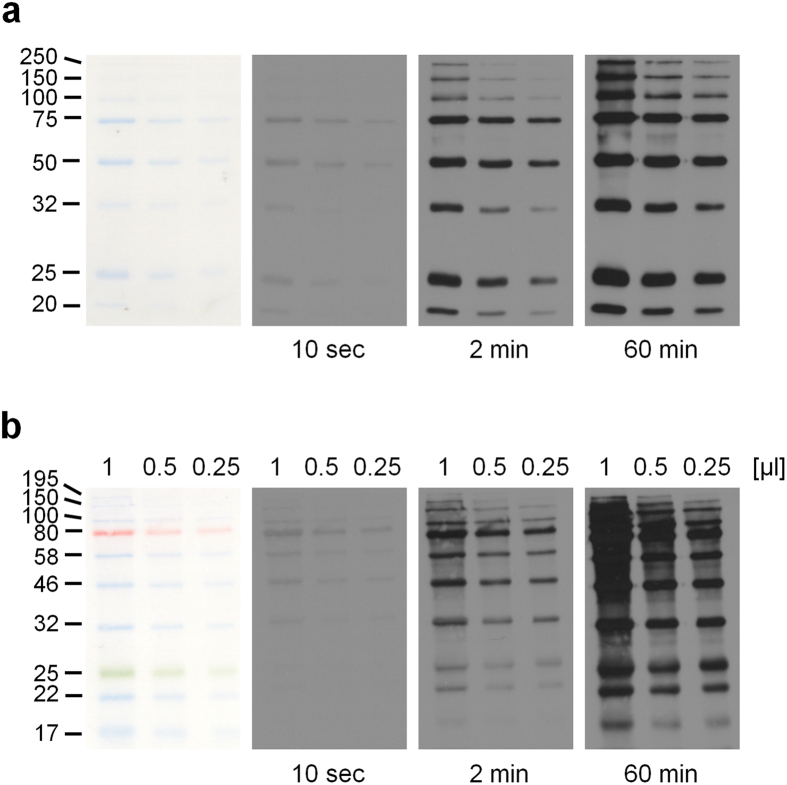
Anti-BLUE and anti-RAINBOW antibodies can be used over a wide range of exposure times. A dilution series from 1 μl to 0.25 μl of Bio-Rad Precision Plus Protein All Blue marker (recommended loading volume by the manufacturer is 10 μl per lane) was detected with HRP-coupled anti-BLUE antibody (0.5 mg/ml, diluted 1:5,000; upper panels). A similar dilution series from 1 μl to 0.25 μl of NEB Color Prestained Broad Range protein standard marker (recommended loading volume by the manufacturer is 5 μl per lane) was detected with HRP-coupled anti-RAINBOW antibody (0.4 mg/ml, diluted 1:5,000; lower panels).

A tool for marker detection in Western blot analysis should be easy to apply and should display the marker simultaneously with the protein of interest over a wide range of exposure times on X-ray film or CCD based imaging systems. Therefore, we coupled the anti-BLUE and anti-RAINBOW antibodies directly to HRP to further facilitate their usage, because the necessity to add two different types of secondary antibody, if the primary antibody species was different from the mouse IgG of the anti-BLUE or anti-RAINBOW, may constitute a potential barrier for their routine application. To determine the exposure time range we tested dilution series of both HRP-coupled antibodies on three different amounts of protein markers and at short to very long exposure times detected by X-ray film (Fig. 3 and Supplementary Figures 8 and 9) and imaging systems such as the ChemiDoc^TM^ (Bio-Rad) and Odyssey (LI-COR) (Supplementary Figure 10). This analysis revealed that both antibodies detected the marker bands over a wide exposure range from seconds to even an hour (Fig. 3). The sensitivity of our antibodies at short exposures yet the lack of background signals at long exposures are further demonstrated in an experiment, where the anti-BLUE antibody was used together with a primary antibody specific for the PPP2R2A (B55α) regulatory subunit of protein phosphatase 2A (PP2A) (Supplementary Figure 11). If particularly weak signals have to be detected with the help of more sensitive ECL reagents, even further reduced concentrations of antibody still resulted in clean marker bands over a wide range of exposures (Supplementary Figure 12). Finally, the colorimetric visibility of many markers is often diminished after stripping and re-probing of the blots. We therefore tested whether re-incubation with anti-BLUE/anti-RAINBOW antibodies would allow marker detection after stripping (Supplementary Figure 13). Indeed, even after the 3^rd^ stripping of the blot the marker bands were recognized by both antibodies with no signal loss compared to previous incubations again showing the excellent detection properties of the anti-BLUE/anti-RAINBOW antibodies.

## Discussion

In summary, we used a tailored immunization approach to develop monoclonal antibodies for the detection of Remazol dye prestained marker proteins. The sequential immunization with hapten-carrier conjugates, in which we exchanged the carrier protein from immunization to immunization, selectively elicited an immune response towards the Remazol dye. Moreover, to generate antibodies cross-reacting with different Remazol dyes we used not only different carrier proteins but also different Remazol dyes in each immunization step. By the sequential immunization with dyes/haptens that share some similarities (such as the aromatic ring system and a vinyl sulfone group) we could indeed induce the maturation of antibodies that did not only cross-react with the dyes used for immunizations but also with other Remazol dyes. Importantly, we achieved such equal cross-reactivity only in mice, which had been immunized with dye-carrier protein complexes of four different colors, while we observed only minor cross-reactivities in mouse sera immunized with the individual colors. Similar sequential immunization strategies have recently been described for the development of cross-reactive antibodies against HIV-1 and influenza^11^,^12^,^13^. In both cases broadly neutralizing antibodies could be generated through the sequential immunization with different virus subtypes.

The exquisite specificity for Remazol-colored proteins makes these antibodies the perfect tools to visualize prestained marker bands side-by-side with the particular protein bands of interest directly on X-ray film or by CCD-camera in Western blot experiments, and ends the anachronistic and error-prone custom of manually marking the prestained marker bands on film exposures. The complete lack of cross-reactivity with unstained proteins allows the usage of these antibodies in combination with any given primary antibody. Furthermore, by using these high-affinity monoclonal antibodies the amount of protein marker required can be reduced by 80–90% compared to the amount recommended by the various vendors. While such small amounts still allow visual monitoring of electrophoretic separation and transfer efficiency, clean and focused marker signals will be obtained over a wide range of exposure times. The Western blot analysis of a series of commercially available molecular weight markers by anti-BLUE and anti-RAINBOW revealed large differences in the quality of these marker preparations some showing high impurities and protein degradation (data not shown). Thus, these antibodies can also be used for the monitoring of molecular weight marker quality.

Finally, we propose that these novel antibodies shall become standard tools for Western blots and the publication of Western blot data, as the current custom of manually marking and showing only small blot sections does not provide the necessary information to reviewers and readers for the judgement of the molecular weight of the depicted proteins. In the future, it should become scientific standard that protein marker bands are displayed side-by-side with the relevant protein bands on the same film/exposure (at least as Supplementary information), to allow the unambiguous validation of the molecular weight of the identified proteins. Last but not least, we imagine the use of these Remazol dye specific antibodies in other methods using Remazol dyes such as protein painting, a method in which surface labeling with Remazol dyes is used to map protein-protein interaction surfaces^8^, mass spectrometry of Remazol stained proteins^9^ and immunoprecipitation-Western blot analysis, in which the (co-) immunoprecipitated proteins are Remazol stained and then detected by the Remazol specific antibodies in the Western blot analysis.

## Materials and Methods

### Purification of prestained marker proteins for immunization

For the Remazol blue stained marker proteins, 500 μl of Precision Plus Protein^TM^ All Blue Standard marker protein mixture (Bio-Rad; identity of proteins and protein concentration/amounts not disclosed by the manufacturer) was separated by preparative 10% SDS-PAGE. The prestained bands corresponding to the 75 kDa, 50 kDa and 25 kDa, respectively, marker proteins were excised from the gel and electro-eluted using a Schleicher&Schuell Elutrap. Electro-eluted proteins were dialyzed for 14 hours at 4 °C against Tris-buffered saline (TBS).

### Remazol dye staining of proteins for immunization

Protein dye staining was performed as described^5^. Briefly, phosphorylase b, bovine serum albumin (BSA), alcohol dehydrogenase (ADH; all three from Sigma), chymotrypsin and lysozyme (both from Serva) were dissolved at 10 mg/ml in 0.15 M sodium chloride. Remazol Brilliant Blue R (RBBR; Sigma), Remazol Brilliant Orange 3R (aka Reactive Orange 16; Sigma), Remazol Golden Yellow RNL, Remazol Brilliant Red F3B, Reactive Black 5 and Remazol Brilliant Violet 5R (all a kind gift of DyStar Textilfarben GmbH, Germany) were dissolved at 10 mg/ml in 10% w/v SDS. 200 μl of each protein solution was mixed with 50 μl of the respective dye solution and 50 μl of 1 M disodium hydrogen phosphate (Na_2_HPO_4_) pH 9.6 and incubated for 20 min at 65 °C. The Remazol dye stained proteins were separated by preparative SDS-PAGE, the bands corresponding to the stained proteins were excised from the gel and the proteins were electro-eluted using a Schleicher&Schuell Elutrap. Electro-eluted proteins were dialyzed for 14 hours at 4 °C against Tris-buffered saline (TBS).

### Immunization of mice and fusion of splenocytes with myeloma cells

Purified Bio-Rad Precision Plus All Blue 75 kDa, 50 kDa and 25 kDa marker proteins as well as purified RBBR stained BSA, ADH and lysozyme were separated by SDS-PAGE to obtain a rough estimate of Bio-Rad marker protein amounts in the purified samples (data not shown). Balb/c mice were consecutively immunized either with approximately 30 μg of RBBR stained BSA, ADH or lysozyme, respectively, or with a similar amount of the 75 kDa, 50 kDa and 25 kDa marker proteins, as judged from the color intensity of the bands on the SDS-PA gel, by injecting the antigens mixed with adjuvant (1:1 ratio) subcutaneously. After three immunizations over the course of five weeks, mouse sera were screened for the presence of dye-specific antibodies by Western blot against Bio-Rad Precision Plus Protein^TM^ All Blue Standard marker proteins. A final boost consisting of a mixture of either the three marker proteins or RBBR-BSA, -ADH and -lysozyme diluted in PBS was injected into the tail vein. Four days after the final immunization splenocytes were fused with the myeloma cell line X63Ag8.653 according to standard protocols using polyethylene glycol^14^. Seven days post fusion, hybridoma supernatants were screened for the presence of antibodies by Western blotting. Positive single clones were obtained by limited dilution of mixed hybridoma clones.

For the anti-Rainbow immunization, two Balb/c mice (M1 & M3) were sequentially immunized with RBBR-BSA, followed by RO16-Chymotrypsin, RGY-Catalase and RF3B-Lysozyme, two other mice (M2 & M4) were sequentially immunized with RF3B-BSA, followed by RGY-catalase, RO16-lysozyme, and RBBR-chymotrypsin, and mouse sera were tested for dye recognition after the fourth immunization. The final boost of M1, prior to splenocyte fusion, was carried out with a mixture of the four dye-protein conjugates.

### Western blotting/Dot blotting

Cells were lysed in pre-heated Laemmli buffer and whole cell proteins were separated by SDS-PAGE, followed by blotting onto a nitrocellulose membrane (Amersham^TM^ Protran^TM^, GE Healthcare; 0.2 μm). For mouse sera and hybridoma supernatant screenings, Precision Plus Protein^TM^ All Blue Standard marker proteins or prestained protein mixtures were separated by preparative SDS-PAGE and blotted onto nitrocellulose membrane. The membranes were blocked with 3% non fat dry milk (NFDM) in PBS-Tween 20 for 1 hour at RT and incubated with primary antibody or mouse sera (diluted 1:500) in 0.5% NFDM/PBS-Tween 20 either o/n at 4 °C or for 2 hours at RT. Incubation with secondary peroxidase conjugated anti-mouse or -rabbit antibody (AffiniPure Goat Anti Mouse or Rabbit IgG (Fc fragment) specific antibody, Jackson ImmunoResearch, 1:5,000) was performed for 1 hour at RT, followed by incubation with ECL Western Blotting reagent (GE Healthcare) as suggested by the manufacturer. For Supplementary Figure 10, a 1:5 dilution of ECL Select Western Blotting reagent (GE Healthcare) in ECL Western Blotting reagent was used. The signals were detected by a Super HR-HA 30 film (Fuji). For Supplementary Figure 13, chemiluminescent signals were detected by the ChemiDoc^TM^ system (Bio-Rad). For detection by fluorescence, the membranes were blocked with 2% BSA in PBS for 1 hour at RT and incubated with primary antibody together with secondary IRDye 680LT goat anti-mouse antibody (LI-COR, 1:20,000) for 1 hour at RT. After washing 3× with PBS-T and once with PBS, images were captured with the Odyssey system (LI-COR). The following commercial antibodies were used: anti Lamin A/C, clone 3A6-4C11 (CST/Enzo Lifesciences/ActiveMotif/Sigma), anti Protein Phosphatase 2A Methylesterase (PME1), clone 8A6-F8 (Biolegend/Millipore), and anti PPP2R2A (PP2A Bα regulatory subunit), clone 2G9 (CST/Millipore/Abcam). Mouse monoclonal antibodies against Cdc55, Net1, and the rabbit polyclonal antibody against PPP2CA (PP2A catalytic subunit) were generated in the authors’ lab. Serum and hybridoma supernatant screening was performed with Immunetics Miniblotter 28 channel units. For dot blots, 1 μl of Precision Plus protein marker (Bio-Rad) was diluted with 4 μl of water and 1 μl of the dilution was spotted onto nitrocellulose and Hybond^TM^-P PVDF membrane (Amersham) (spot 1). 1 μl of a serial dilution of RBBR (corresponding to 10 μg, 1 μg, 0.1 μg, and 0.01 μg) was spotted onto each membrane (spots 2–5), and the membranes were treated as described above. Although easily visible with the naked eye on the membrane, colorimetric scans of pre-stained markers and proteins were adjusted for brightness and contrast to achieve presentable figure images.

### Western blot stripping

After ECL detection, membranes were washed 3× in PBS-Tween and incubated in freshly prepared stripping solution (0.06 M Tris pH 6.8, 2% SDS, 0.1 M β-mercaptoethanol) for 30 min at 52 °C with gentle agitation. After washing 6× in PBS-Tween, the membranes were re-incubated with HRP-coupled anti-BLUE or anti-RAINBOW antibody, respectively, for one hour at RT, and signals were developed with ECL Western Blotting reagent.

### Ethics Statement

The maintenance of mice and experimental procedures have been conducted according to the Austrian Animal Experiments Act and have been approved by the Austrian Federal Ministry of Science and Research (GZ 66.009/0040-II/3b/2011 and GZ 66.009/0013-II/10b/2010) and the animal experiments ethics committee of the Medical University of Vienna.

## Additional Information

**How to cite this article**: Schüchner, S. *et al*. Anti-RAINBOW dye-specific antibodies as universal tools for the visualization of prestained protein molecular weight markers in Western blot analysis. *Sci. Rep.* **6**, 31363; doi: 10.1038/srep31363 (2016).

## Supplementary Material

Supplementary Information
