# Supplementary material for: Anti-RAINBOW dye-specific antibodies as universal tools for the visualization of prestained protein molecular weight markers in Western blot analysis
Source: Sci Rep. 2016 Aug 17;6:31363. doi: 10.1038/srep31363 (PMC4987645; doi:10.1038/srep31363)
Supplement: Supplementary Information [file srep31363-s1.pdf]

Supplementary information for Schüchner et al. "Anti-RAINBOW dye-specific antibodies as universal tools for the visualization of prestained protein molecular weight markers in Western blot analysis"

Stefan Schüchner, Peter Andorfer, Ingrid Mudrak, and Egon Ogris

Page

Supplementary figures and legends **2**

Supplementary Figure 1: Mouse sera preferentially detect prestained proteins 2

Supplementary Figure 2: Mice develop antibodies recognizing prestained marker proteins 3

Supplementary Figure 3: anti-BLUE monoclonal antibody does not detect free RBBR 4

Supplementary Figure 4: anti-BLUE monoclonal antibody does not interfere with the detection of proteins by other antibodies 5

Supplementary Figure 5: Chemical structures of Remazol dyes 6

Supplementary Figure 6: Rainbow immunized mouse sera show cross-reactivity with Remazol dyes not used for immunization 7

Supplementary Figure 7: anti-RAINBOW monoclonal antibody detects the blue pre-stained Precision Plus WesternC protein marker bands 8

Supplementary Figure 8: anti-RAINBOW monoclonal antibody can be used over a wide range of exposure times 9

Supplementary Figure 9: anti-BLUE monoclonal antibody can be used over a wide range of exposure times 10

Supplementary Figure 10: anti-BLUE or anti-RAINBOW monoclonal antibodies can be used with camera-based detection systems 11

Supplementary Figure 11: Simultaneous detection of prestained protein marker and PP2A Bα subunit 12

Supplementary Figure 12: anti-BLUE monoclonal antibody can be used over a wide range of exposure times with more sensitive ECL reagents 13

Supplementary Figure 13: Pre-stained protein markers can be reprobed with anti-BLUE or anti-RAINBOW monoclonal antibodies after stripping 14

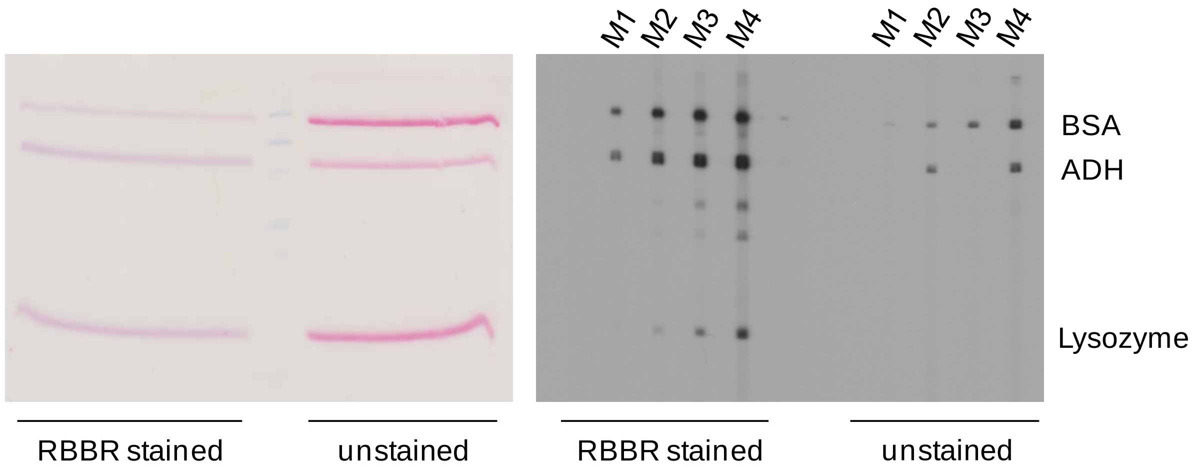

Supplementary Figure 1: Mouse sera immunized with RBBR-prestained proteins preferentially detect RBBR-stained proteins but show minor cross-reactivity against the carrier proteins. Mixtures containing approximately 2 µg of each protein (BSA, ADH, and lysozyme), either unstained or prestained with RBBR, were separated by 15% SDS-PAGE, transferred to nitrocellulose membrane and stained with Ponceau S (left panel). The membrane was then incubated as described in Materials & Methods with the indicated mouse sera (right blot). The mice had been immunized first with RBBR-BSA (6 weeks prior to immune serum), followed by a boost with RBBR-ADH (4 weeks prior to immune serum) and a boost with RBBR-lysozyme (10 days prior to immune serum).

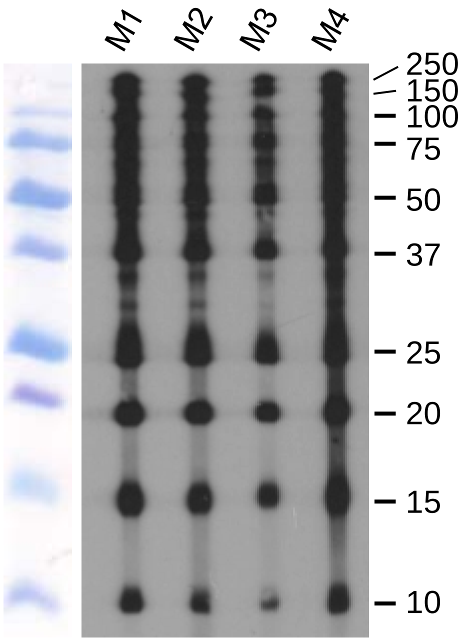

Supplementary Figure 2: Immunization with three RBBR stained proteins induces a general immune response against prestained marker proteins. Immune sera of four mice (M1-M4) sequentially immunized with RBBR-BSA, RBBR-ADH and RBBR-lysozyme were tested by Western blot for the presence of reactive antibodies against Bio-Rad Precision Plus All Blue marker proteins separated by preparative 15% SDS-PAGE.

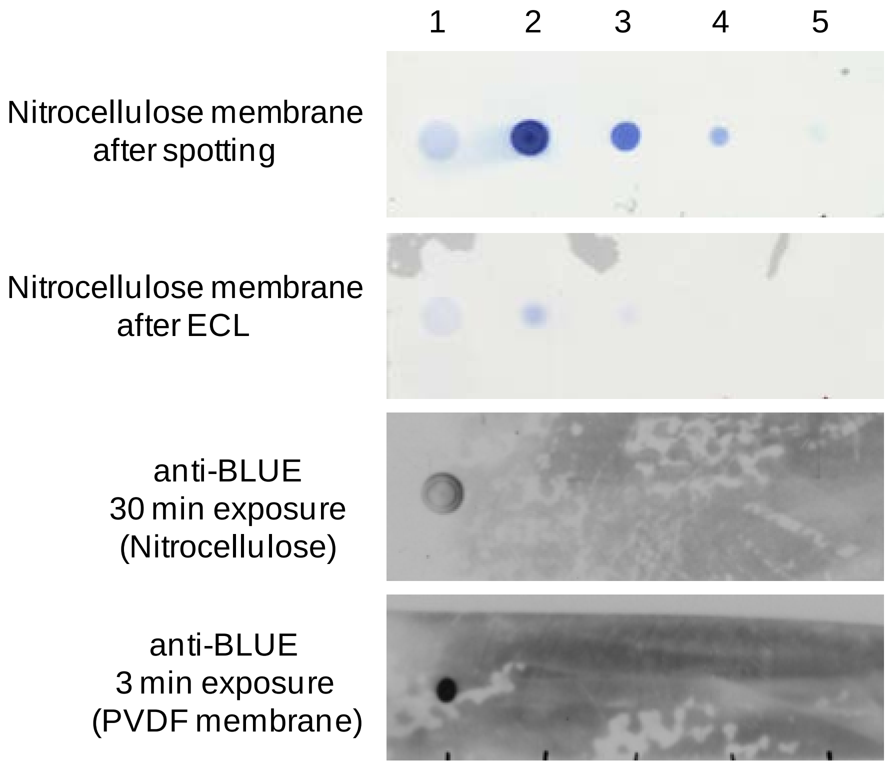

Supplementary Figure 3: anti-BLUE monoclonal antibody does not detect free RBBR. 0.2 µl of prestained Bio-Rad Precision Plus All Blue protein marker (lane 1) or decreasing amounts of free RBBR dye (lane 2: 10 µg, lane 3: 1 µg, lane 4: 0.1 µg, lane 5: 0.01 µg) were spotted onto nitrocellulose or PVDF membrane and incubated with anti-BLUE monoclonal antibody.

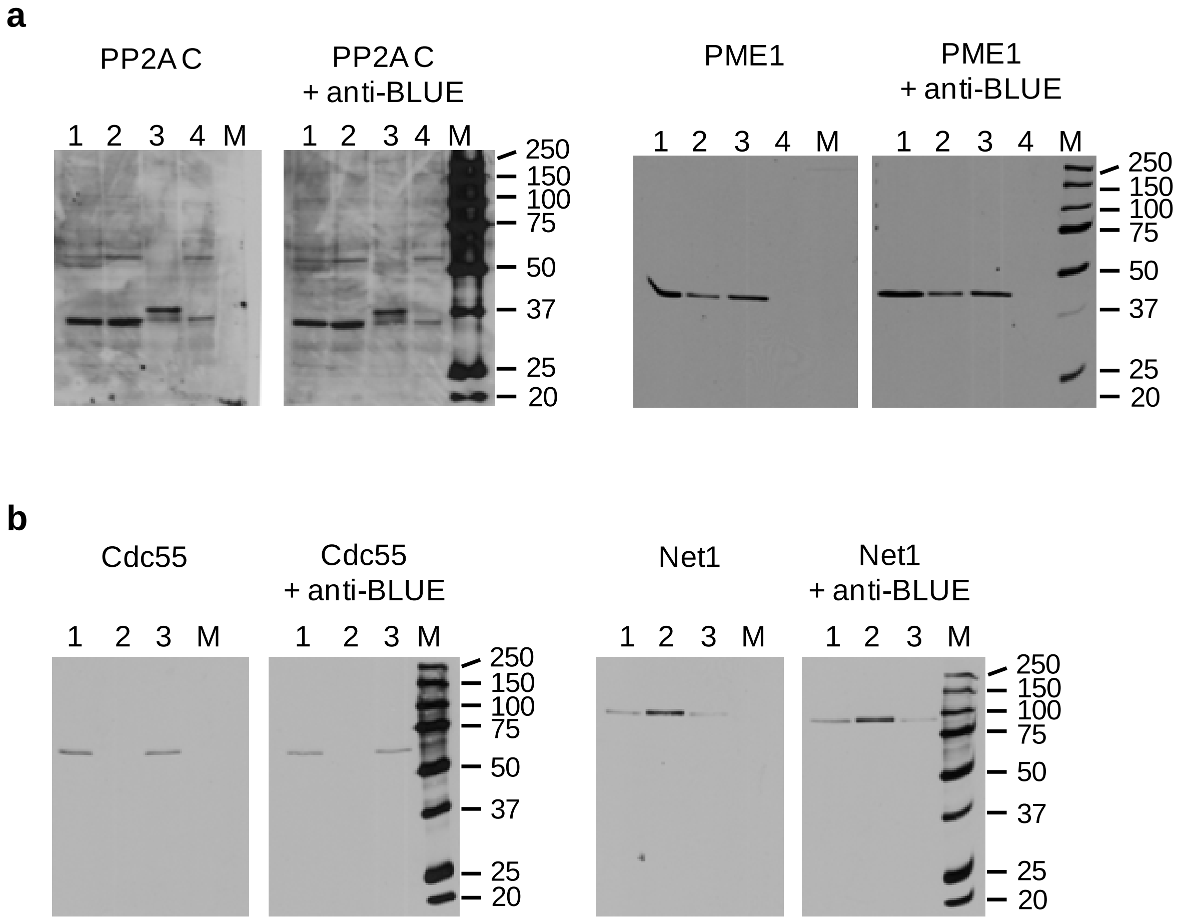

Supplementary Figure 4: anti-BLUE monoclonal antibody does not interfere with the detection of proteins by other antibodies. For each of the indicated antibodies, one of two identical blots containing mammalian or yeast lysates was incubated with the respective antibody alone (left panels) or in combination with anti-BLUE (right panels). **a)** Western blots with mammalian lysates were incubated with a rabbit polyclonal antibody against PP2AC (left panels) or with a mouse monoclonal antibody against PME1 (right panels); lane 1: U2OS; lane 2: NIH3T3; lane 3: NIH3T3 myc-PP2A Cα; lane 4: MEF PME1$^{-/-}$; lane M: Bio-Rad Precision Plus Protein All Blue (1 µl). **b)** Western blots with yeast lysates were incubated with monoclonal antibodies against Cdc55 (left panels) or Net1 (right panels). Lane 1: BY4741 4H3-2HA-Net1$^{1\text{-}600}$; lane 2: *cdc55Δ* 4H3-2HA-Net1$^{1\text{-}600}$; lane 3: *ppe1Δ* 4H3-2HA-Net1$^{1\text{-}600}$; lane M: Bio-Rad Precision Plus Protein All Blue (1 µl).

Remazol Brilliant Blue R

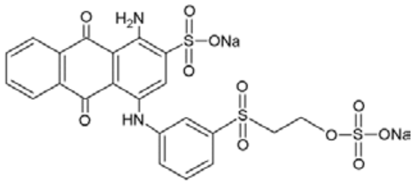

Remazol Orange 16

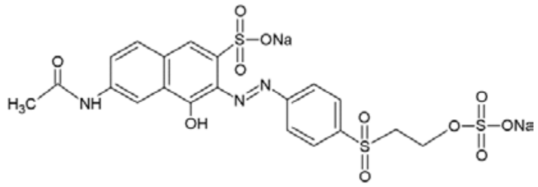

Remazol Brilliant Red F3B

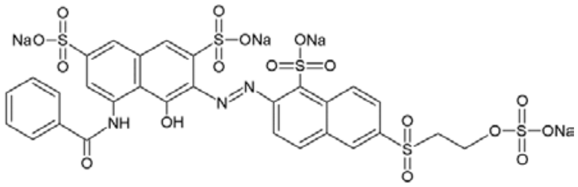

Remazol Golden Yellow RNL

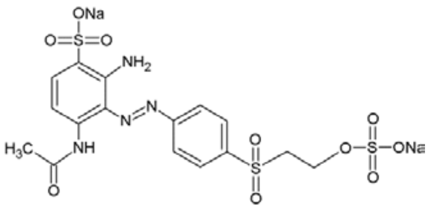

Remazol Brilliant Violet

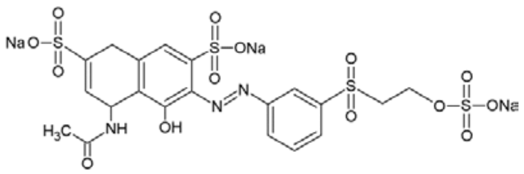

Remazol Reactive Black 5

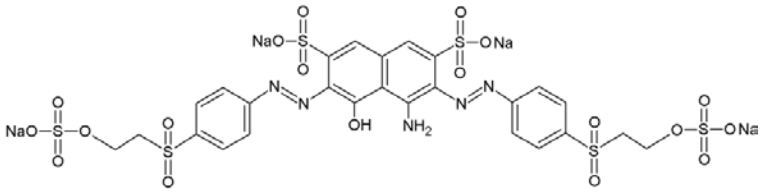

Supplementary Figure 5: Chemical structures of Remazol dyes. Structures were drawn using the ChemSketch software (ACD Labs).

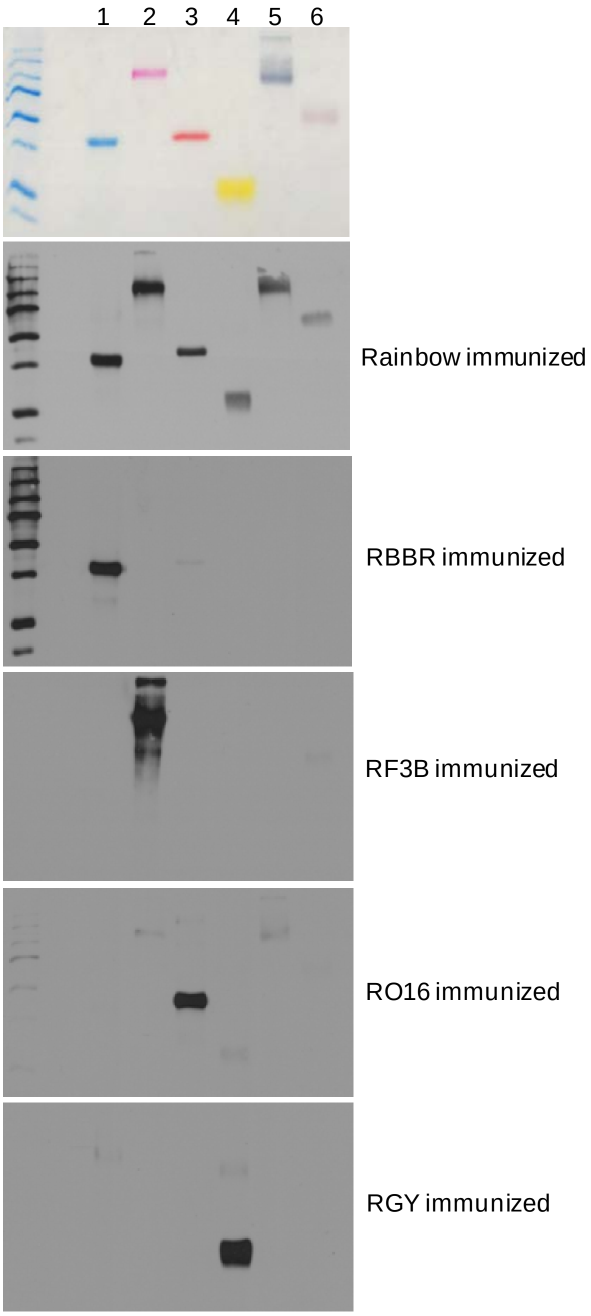

Supplementary Figure 6: Rainbow immunized mouse sera show cross-reactivity with Remazol dyes not used for immunization. Sera of mice immunized with either RBBR, or RF3B, or RO16, or RGY (performed as described in Materials & Methods for the RBBR immunization), or sequentially with all four dyes (Rainbow immunization as described in Materials & Methods) were tested for the detection of RBBR-ADH (blue, lane 1), RF3B-phosphorylase b (red, lane 2), RO16-ADH (orange, lane 3), RGY-γ-globulin light chain (yellow, lane 4), RB5-phosphorylase b (black, lane 5), and RV5R-γ-globulin heavy chain (violet, lane 6). While also the single color immunized mouse sera weakly recognized some of the other dyes (e.g. detection of several dyes including the BioRad Precision marker bands by the RO16 serum), only the rainbow immunized serum displayed strong cross-reactivity with all six dyes. 5x more Biorad marker and 10x more of the Remazol stained proteins were loaded on the colorimetric blot than on the blots incubated with mouse sera.

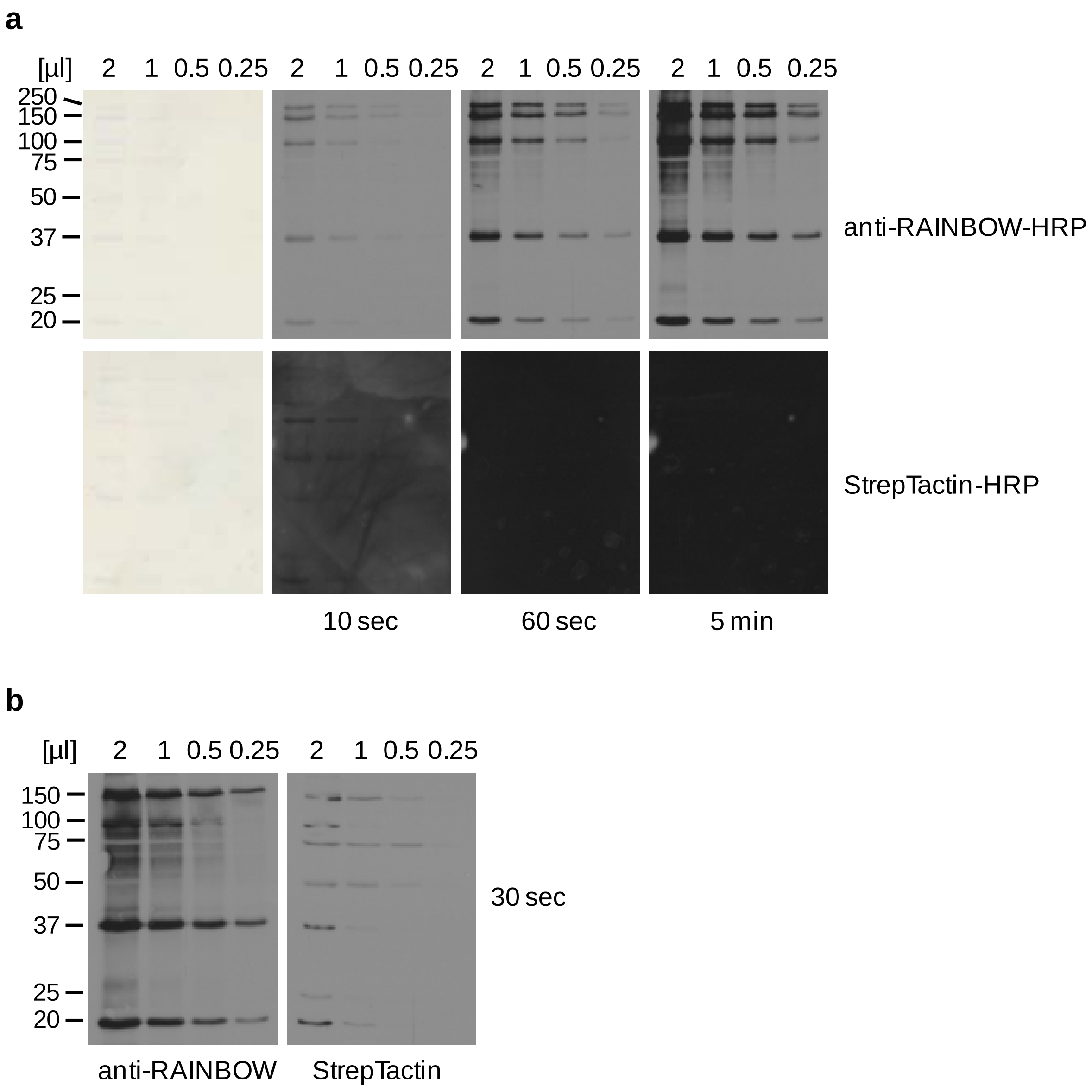

Supplementary Figure 7: anti-RAINBOW monoclonal antibody detects the blue pre-stained Precision Plus WesternC protein marker bands. a) A dilution series from 2µl to 0.25µl of Bio-Rad Precision Plus Protein WesternC^{TM} marker (recommended loading volume by the manufacturer is 10µl per lane) was detected with HRP-coupled anti-RAINBOW antibody (0.4mg/ml, 1:10000) or with StrepTactin-HRP (1:10000). For anti-RAINBOW-HRP the membrane was blocked in 3% NFDM in PBST, and the antibody was diluted in 0.5% NFDM/PBST. For StrepTactin-HRP, the membrane was blocked in 3% BSA in TBS, and Strep-Tactin-HRP was diluted in 1% BSA/PBS (as recommended by the manufacturer). **b)** A dilution series from 2µl to 0.25µl of Bio-Rad Precision Plus Protein WesternC^{TM} marker was detected with HRP-coupled anti-RAINBOW antibody (0.4mg/ml, 1:10000) or with StrepTactin-HRP (1:10000) after blocking with 3% NFDM/PBST.

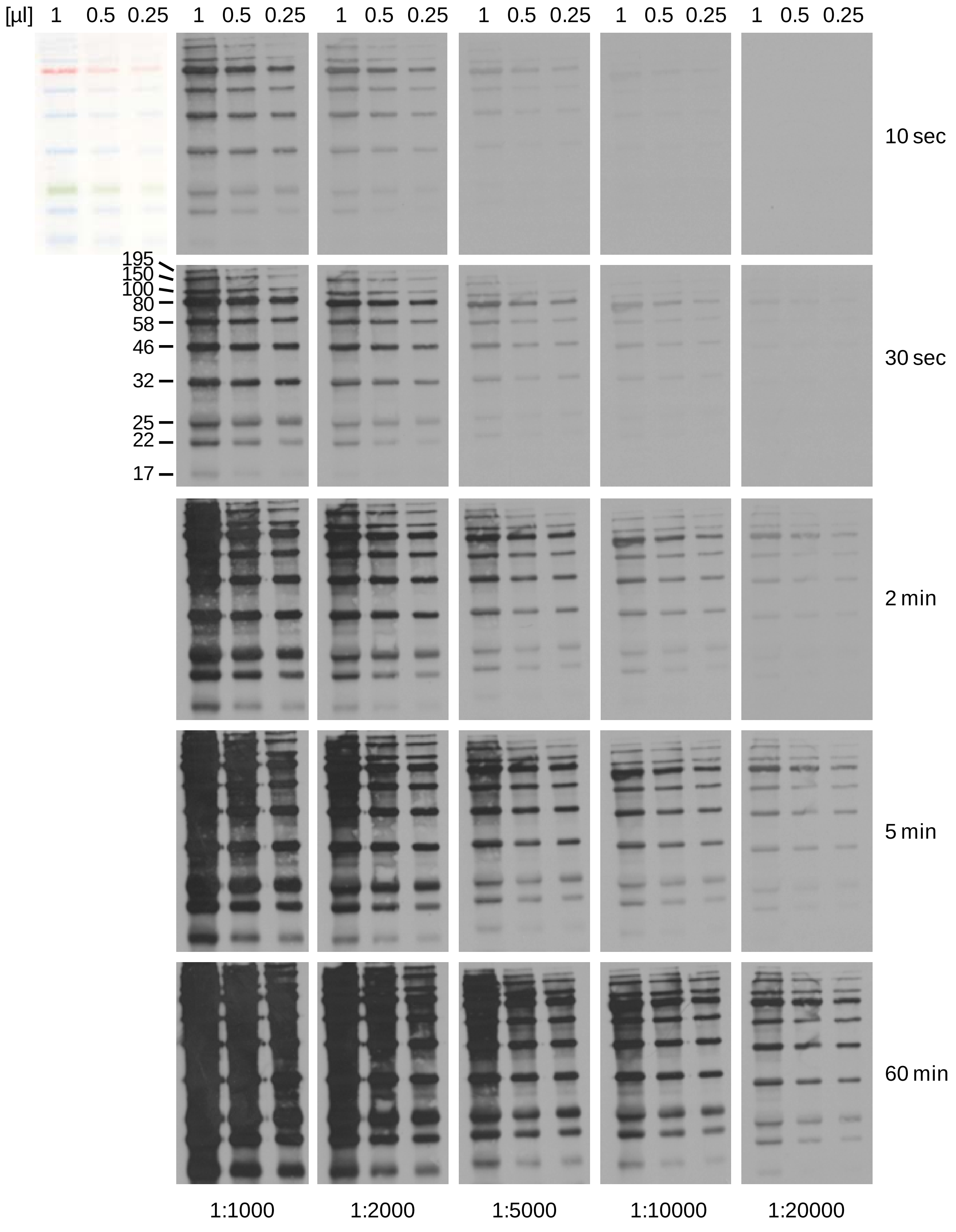

Supplementary Figure 8: anti-RAINBOW antibody can be used with high sensitivity and over a wide range of dilutions and exposure times. A dilution series from 1µl to 0.25µl of NEB Color Prestained Broad Range protein standard marker (recommended loading volume by the manufacturer is 5µl per lane) was detected with HRP-coupled anti-RAINBOW antibody (0.4mg/ml, serially diluted from 1:1,000 up to 1:20,000). Chemiluminscence was detected with ECL Western Blotting Detection Reagents (GE Healthcare).

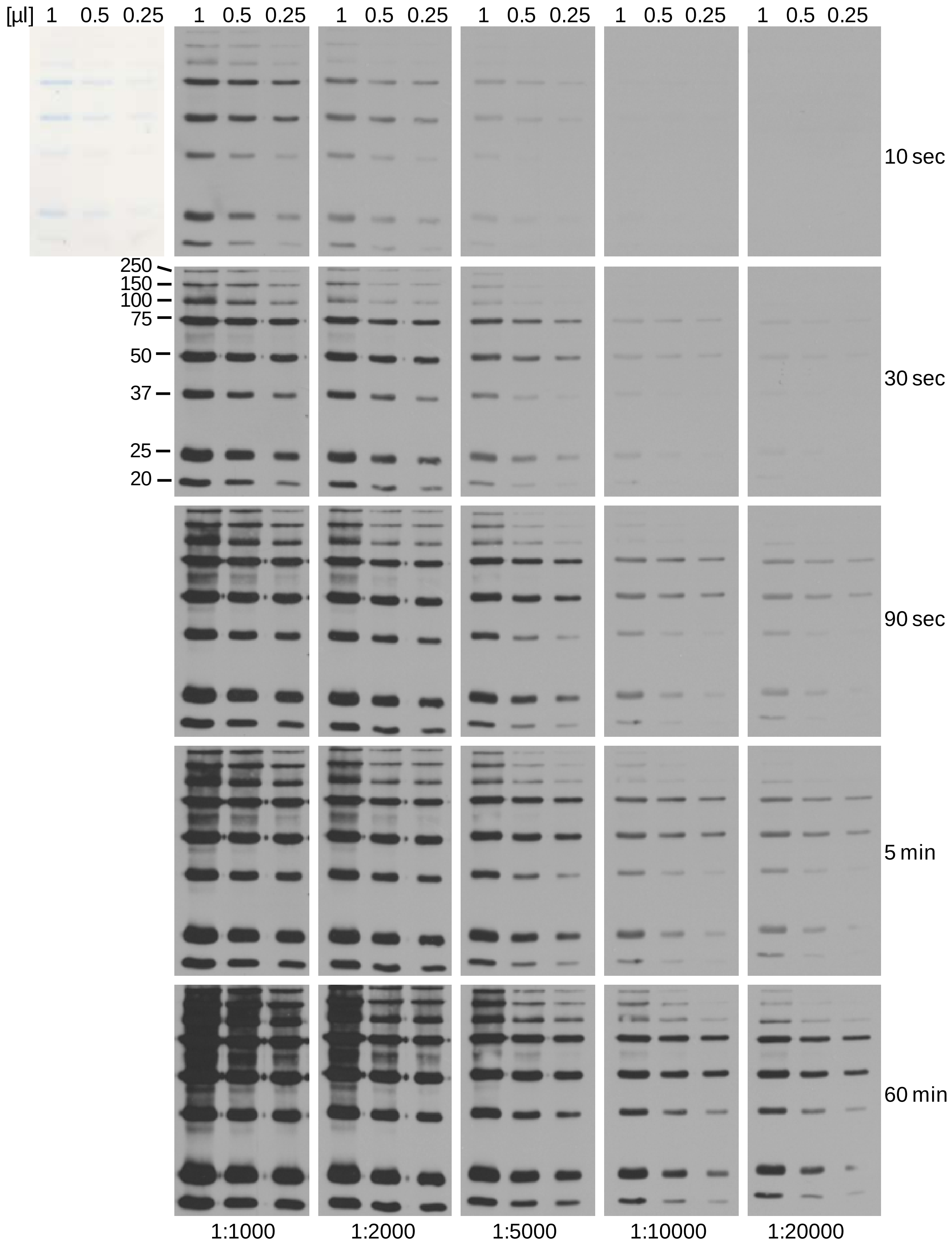

Supplementary Figure 9: anti-BLUE antibody can be used with high sensitivity and over a wide range of dilutions and exposure times. A dilution series from 1µl to 0.25µl of Bio-Rad Precision Plus Protein All Blue marker (recommended loading volume by the manufacturer is 10µl per lane) was detected with HRP-coupled anti-BLUE antibody (0.5mg/ml, serially diluted from 1:1,000 up to 1:20,000). Chemiluminscence was detected with ECL Western Blotting Detection Reagents (GE Healthcare).

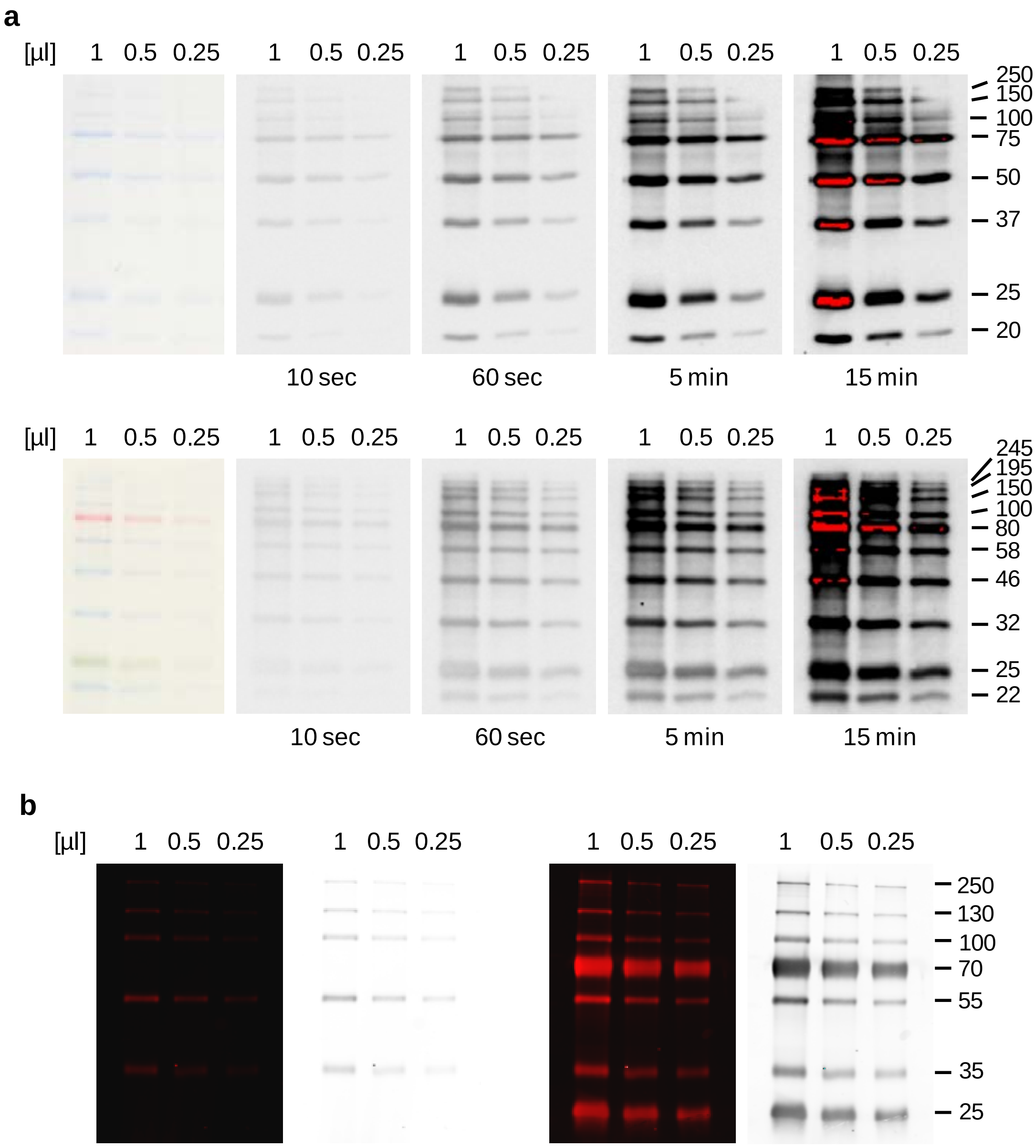

Supplementary Figure 10: anti-BLUE or anti-RAINBOW monoclonal antibodies can be used with camera-based detection systems. a) Dilution series of Bio-Rad Precision Plus Protein All Blue marker (upper panels) or NEB Color Prestained Broad Range marker (lower panels) were detected with HRP-coupled anti-BLUE antibody (0.5mg/ml, 1:10,000) or HRP-coupled anti-RAINBOW antibody (0.4mg/ml, 1:20,000), using ECL Western Blotting Detection Reagents and the Chemidoc imaging system (Bio-Rad). **b)** Detection of a dilution series of the Thermo Scientific PageRuler Plus Prestained Protein Ladder by anti-RAINBOW antibody using the Odyssey imaging system (LI-COR). The left panel shows the direct fluorescence image of the Western blot membrane, displaying only the blue-color prestained marker bands in the 700nm fluorescence channel but not the orange-red prestained marker bands (none of the bands was visible in the 800nm channel). The membrane was then incubated with anti-RAINBOW antibody (1:10,000) together with IRDye 680LT goat anti-mouse antibody (LI-COR; 1:20,000), which detected all marker bands (second fluorescence image, right panel). For better contrast, the original color-coded images were adjusted for brightness and contrast and are also displayed as grey-tone images (unadjusted).

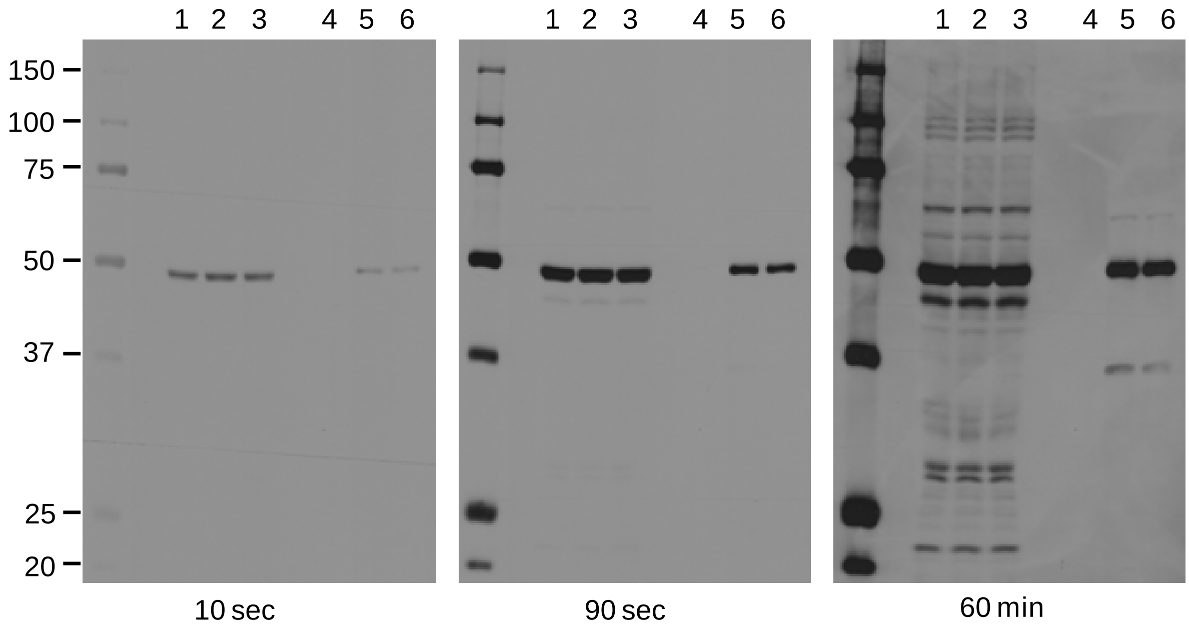

Supplementary Figure 11: Simultaneous detection of prestained protein marker and PP2A Bα subunit. Whole cell lysates of mouse fibroblasts expressing HA-tagged PP2A (lanes 2 and 3) or mock transfected control cells (lane 1) were subjected to an anti-HA tag immunoprecipitation. Lysates and IPs (lanes 4-6) were analysed for PP2A Bα subunit expression/association by incubation with PP2A Bα specific monoclonal antibody 2G9 at 4°C o/n, followed by incubation with anti-BLUE (1:5,000) together with HRP-coupled secondary anti-mouse antibody for one hour at RT. Chemiluminscence detection was performed with ECL Western Blotting Detection Reagents (GE Healthcare).

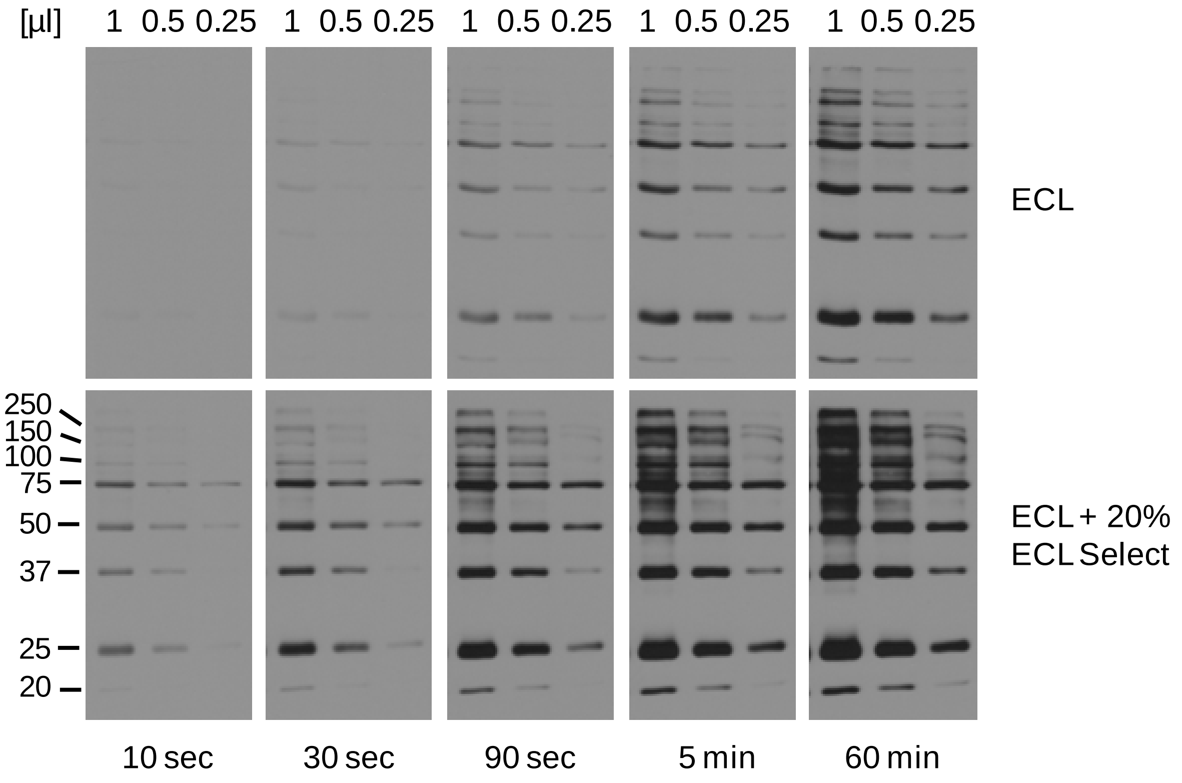

Supplementary Figure 12: anti-BLUE antibody can be used over a wide range of exposure times with more sensitive ECL reagents. A dilution series from 1µl to 0.25µl of Bio-Rad Precision Plus Protein All Blue marker (recommended loading volume by the manufacturer is 10µl per lane) was detected with HRP-coupled anti-BLUE antibody (0.5mg/ml, 1:10,000). Chemiluminscence detection was performed with either ECL Western Blotting Detection Reagents (upper panels) or with ECL Select Western Blotting Detection Reagents diluted 1:5 in ECL Western Blotting Detection Reagents (lower panels).

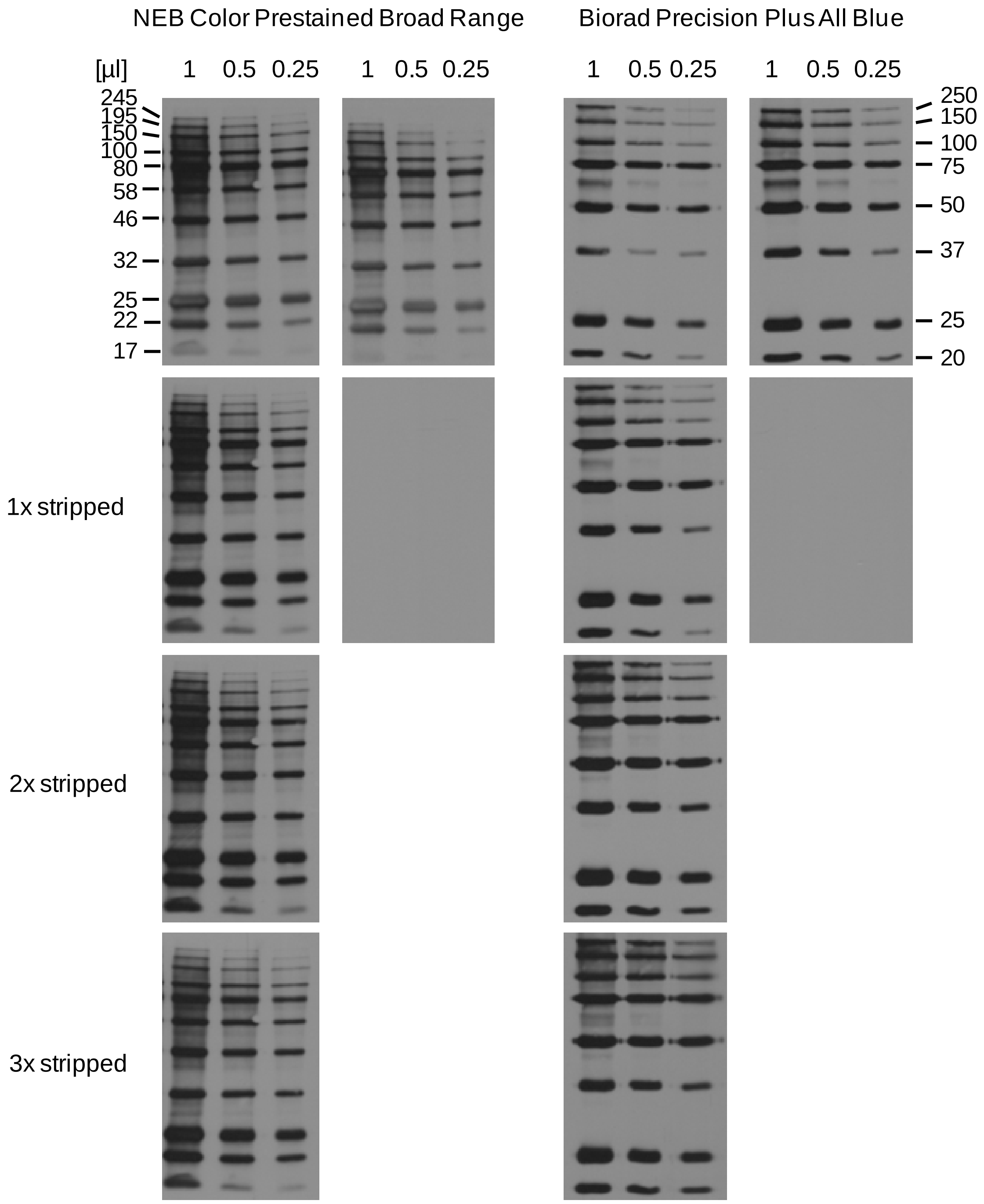

Supplementary Figure 13: Pre-stained protein markers can be reprobed with anti-BLUE or anti-RAINBOW monoclonal antibodies after stripping. Dilution series of NEB Color Prestained Broad Range marker (left panels) or Bio-Rad Precision Plus Protein All Blue marker (right panels) were detected with HRP-coupled anti-RAINBOW (0.4mg/ml, 1:2,000, left panels), or HRP-coupled anti-BLUE antibody (0.5mg/ml, 1:2,000, right panels). After chemiluminescence detection, the membranes were stripped as described in Materials and Methods. One membrane each was then re-incubated with the respective antibody (1x stripped, left hand blots), the other one just washed (1x stripped, right hand blots). After chemiluminescence detection, the membranes were stripped again, re-incubated a second time with the repective antibodies, and finally stripped and re-incubated for a third time.
